# Occurrence Dynamics and Prediction of Coleoptera and Lepidoptera in China Using Multiple Machine Learning Models

**DOI:** 10.3390/insects17090906

**Published:** 2026-08-29

**Authors:** Hong Sun, Jiaqi Zhang, Xiumei Mo, Chenshu Zhang, Ziqi Wang, Jixia Huang

**Affiliations:** 1 Center for Biological Disaster Prevention and Control, National Forestry and Grassland Administration, Shenyang 110034, China; sunhongcaf@163.com (H.S.);; 2 Key Laboratory of National Forestry and Grassland Administration on Invasive Alien Species Prevention and Control, National Forestry and Grassland Administration, Shenyang 110034, China; 3 State Key Laboratory of Efficient Production of Forest Resources, Beijing Forestry University, Beijing 100083, China; 18800194896@163.com (J.Z.);; 4 Key Laboratory of Land Surface Pattern and Simulation, Chinese Academy of Science, Beijing 100101, China

**Keywords:** coleoptera, lepidoptera, climatic factors, machine learning, insect population dynamics

## Abstract

The temporal dynamics of forest-associated insects are closely associated with climatic variability, and identifying their temporal patterns and climatic associations is important for understanding insect population dynamics. In this study, automatically recorded Coleoptera and Lepidoptera in Rui’an City, Zhejiang Province, China, were selected as the target insect groups. Based on automatic insect monitoring and meteorological data, the temporal characteristics of recorded insect abundance and their associations with climatic factors were analyzed. Five machine learning models, including Multiple Linear Regression, K-Nearest Neighbors, Random Forest, Support Vector Regression, and Generalized Additive Model, were then developed and compared for predicting recorded insect abundance. The results showed that Coleoptera exhibited periodic fluctuations with cycles of approximately 6–20 days, whereas Lepidoptera displayed persistent seasonal variation. Air temperature, specific humidity, and shortwave radiation were significantly and positively correlated with recorded insect abundance, while wind speed showed a weak negative correlation. Among the five models, Random Forest achieved the best prediction performance, with MAE, RMSE, and R^2^ values of 1.37, 4.85, and 0.82 for Coleoptera and 0.57, 1.16, and 0.84 for Lepidoptera, respectively. Independent-year validation using 2023 data showed reduced predictive performance compared with random validation, indicating limited temporal generalization under the available observation period. These findings demonstrate that integrating temporal characteristics with climatic factors can support the prediction of automatically recorded insect abundance, while longer-term monitoring data are needed to further evaluate model robustness and temporal transferability.

## Simple Summary

Forest insects play important roles in forest ecosystems, while some species can also cause substantial ecological and economic damage when their populations reach damaging levels. Their abundance and activity are strongly influenced by weather conditions, but predicting insect population dynamics remains challenging because insect groups differ in their temporal patterns and responses to environmental factors. In this study, we investigated two insect groups recorded by an automatic forest insect monitoring system, Coleoptera and Lepidoptera, using monitoring data and weather records collected in Rui’an City, Zhejiang Province, China. We first analyzed the temporal dynamics of the recorded insect groups and examined their associations with climatic factors. We then compared five different prediction methods to determine which provided the most accurate predictions of recorded insect abundance. The results showed that air temperature, specific humidity, and solar radiation were significantly associated with the recorded abundance of both insect groups. Among the five methods, Random Forest produced the highest predictive accuracy under the internal validation framework. These findings provide a practical approach for analyzing and predicting the temporal dynamics of automatically recorded insect groups and may provide useful information for insect population forecasting.

## 1. Introduction

Forest-associated insects represent an important biological disturbance affecting forest ecosystem stability and can have significant impacts on forest resource security, ecosystem functioning, and forestry economic development [1]. In recent years, under the combined influence of global climate change and increasing human activities, some economically important insect species have shown changes in their distribution, phenology, and population dynamics [2,3]. Severe infestations by economically important forest insect species can cause growth decline and large-scale tree mortality, reduce forest carbon sequestration capacity, disrupt regional ecological balance, and exert long-term impacts on water conservation and biodiversity protection [4,5,6]. Compared with other natural disasters, severe infestations of economically important forest insects may be difficult to detect at an early stage and may require prolonged management efforts. Once severe infestations occur, management costs increase substantially [7]. Therefore, improving monitoring, early warning, and accurate prediction of economically important forest insect species has become an important research focus in forest resource management and ecological security.

Among forest-associated insects, Coleoptera and Lepidoptera comprise taxonomically and ecologically diverse groups that include species with different feeding habits and ecological roles. Some species within these groups are recognized as economically important forest insects, whereas others may have limited or no direct significance for forest damage. In forest ecosystems, phytophagous lepidopteran larvae commonly feed on foliage and young shoots, whereas phytophagous beetles may utilize a wider range of plant tissues, including foliage, roots, bark, phloem, and wood, depending on the taxonomic group and life stage [8,9,10]. Therefore, the ecological and economic significance of individual species cannot be inferred solely from their higher-level taxonomic classification. Due to their diverse life histories, population dynamics, and responses to climatic conditions [11,12,13], these insect groups may exhibit substantial temporal variation in forest environments. In recent years, climate change has raised concerns about changes in the distribution and activity of some economically important forest insect species. However, compared with well-documented major forestry recorded insect-related problems, including pine caterpillars (*Dendrolimus* spp.), fall webworms (*Hyphantria cunea*), and Pine Wilt Disease caused by the pine wood nematode (*Bursaphelenchus xylophilus*) [14,15,16], relatively few studies have examined the temporal dynamics and climatic associations of automatically recorded Coleoptera and Lepidoptera at the group level.

The activity, development, and population dynamics of Coleoptera and Lepidoptera are closely associated with climatic conditions. Meteorological factors such as temperature, humidity, wind speed, and solar radiation can influence insect development, activity, reproduction, and dispersal [17,18,19]. Suitable climatic conditions can improve overwintering survival and reproductive efficiency, whereas extreme climate events may alter the spatial and temporal distribution patterns of insect populations [16]. As climate warming intensifies, the suitable habitats of some economically important insect species are gradually expanding toward higher latitudes and elevations, increasing the potential risks faced by forest ecosystems [20]. At the same time, complex interactions often exist among different meteorological factors, resulting in distinct nonlinear responses and stage dependent differences in the responses of insect populations to climate change [21]. Traditional analyses based on single factors are insufficient to fully reveal the complex response relationships involved in insect population dynamics. Therefore, conducting comprehensive analyses of multiple meteorological factors and revealing the temporal patterns and climatic associations of recorded insect groups are of great importance for improving understanding and prediction of forest insect dynamics.

To better understand insect population dynamics and improve forecasting capabilities, a variety of approaches have been developed for monitoring and predicting forest insects. Traditional approaches include field population monitoring, empirical statistical models, and phenology-based models, which use observed population abundance, climatic variables, and developmental thresholds to characterize seasonal emergence and population changes [22]. These methods are useful for identifying seasonal activity patterns and estimating the timing of insect development, but their predictive performance may be limited when insect–environment relationships are nonlinear or when climatic conditions vary substantially among years [23]. Remote sensing has also been increasingly used to detect forest damage and habitat conditions associated with insect activity, although its effectiveness may depend on the spatial and temporal resolution of the available imagery and on the ability to distinguish insect-induced vegetation changes from other disturbances. More recently, machine learning methods have been applied to forest insect prediction because of their ability to capture nonlinear relationships and interactions among multiple environmental variables [24,25]. However, most existing studies have focused primarily on improving prediction accuracy, while comprehensive analyses combining temporal variation characteristics, climatic associations, and model comparison remain relatively limited. Moreover, temporal dependence and interannual variation may lead to optimistic estimates when conventional random data partitioning is used for model evaluation. Therefore, evaluating both predictive performance and temporal generalization is important when developing machine-learning-based approaches for forest insect prediction.

In this study, automatically recorded monitoring data of Coleoptera and Lepidoptera together with corresponding meteorological data from Rui’an City, Zhejiang Province, China, were used to investigate the temporal dynamics and climatic associations of these two insect groups. Because the monitoring system provided group-level records rather than species-level identification, the study focused on recorded abundance and temporal dynamics and did not infer species-specific recorded insect status, economic significance, or forest damage.

Specifically, we aimed to: (1) characterize the periodic and seasonal variation patterns of the two recorded insect groups; (2) quantify their associations with climatic factors, including temperature, humidity, wind speed, and shortwave radiation; and (3) compare the predictive performance and temporal generalization of five machine-learning models, including Multiple Linear Regression (MLR), Generalized Additive Model (GAM), K-Nearest Neighbors (KNN), Support Vector Regression (SVR), and Random Forest (RF). Based on the ecological characteristics of insect population dynamics and the nonlinear relationships that may exist between insects and climatic factors, we proposed three hypotheses: (1) the two recorded insect groups exhibit distinct periodic and seasonal variation patterns; (2) climatic factors are significantly associated with the temporal variation in their recorded abundance, with the strength of associations differing between the two groups; and (3) nonlinear machine learning models, particularly Random Forest, provide better predictive performance than conventional linear or additive models, but model performance decreases when evaluated under temporally independent conditions. By testing these hypotheses, this study aims to provide a quantitative framework for analyzing and predicting the temporal dynamics of automatically recorded forest insects while clarifying the limitations associated with group-level monitoring data.

## 2. Materials and Methods

### 2.1. Study Area

Rui’an City is located in the southeastern coastal region of Zhejiang Province, China, and is administratively affiliated with Wenzhou City. It is situated between 120°10′ E and 121°15′ E longitude and between 27°40′ N and 28°00′ N latitude. The study area has a subtropical monsoon climate characterized by warm and humid conditions with synchronized rainfall and heat. The annual mean temperature is approximately 17.9 °C. The forest coverage rate of Rui’an City is approximately 49.1%, with *Pinus massoniana* forests, broadleaved forests, and mixed coniferous-broadleaved forests being the major forest types in the region. The region is rich in forest resources, providing suitable habitats for forest-associated insects, including Coleoptera and Lepidoptera. Influenced by the subtropical monsoon climate, meteorological factors such as temperature, humidity, and radiation show strong seasonal variation, providing a good basis for analyzing the relationship between forest recorded insects and climate.

### 2.2. Data

#### 2.2.1. Automatic Insect Monitoring Data

**Figure 1:**
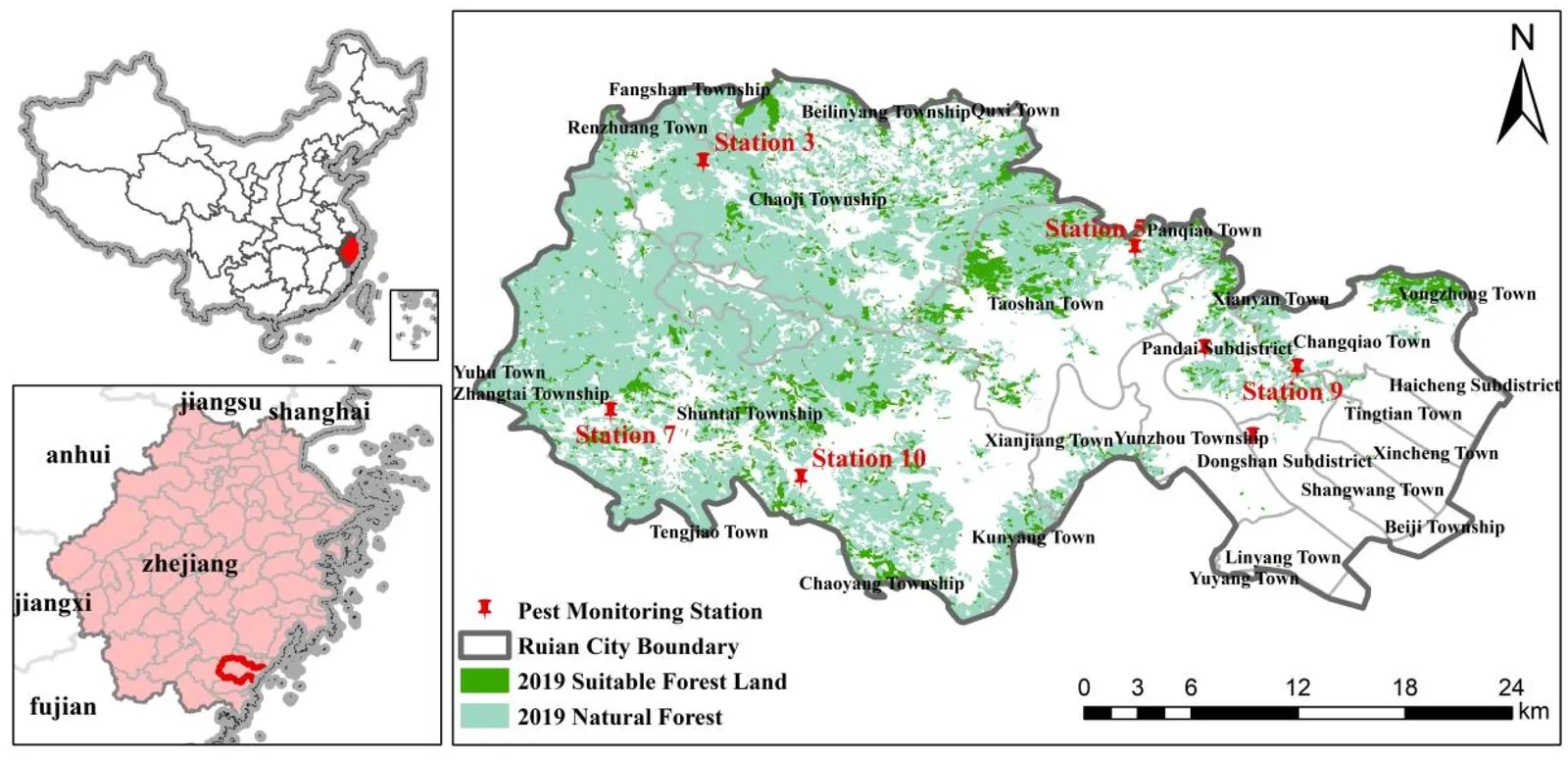
Distribution of light trap monitoring stations for forest recorded insects.

Forest recorded insect data were obtained from automatic forest recorded insect monitoring stations in Rui’an City, Zhejiang Province (Figure 1). A total of 10 automatic insect monitoring stations equipped with light traps were deployed across forested areas in different parts of Rui’an City. The monitoring system employed Internet of Things technology, high-resolution industrial cameras, and machine-learning-based image recognition technology, enabling the automatic identification and counting of multiple forest recorded insect types. Based on the historical monitoring records and data completeness, two major insect groups recorded by the automatic insect monitoring system, Coleoptera and Lepidoptera, were selected as the target groups in this study. The monitoring period was from 1 December 2021 to 30 September 2023, with a daily temporal resolution.

Through automatic trapping and high-resolution image acquisition, the system extracted image features based on machine learning, automatically classified the recorded insects into predefined insect groups, and recorded their abundance. According to the information provided for the monitoring system, its reported recognition accuracy exceeded 85%. The system simultaneously recorded environmental variables including air temperature, air humidity, wind speed, wind direction, and light intensity. During the study period, Stations 4, 6, and 8 experienced long-term equipment failure or missing data, resulting in discontinuous observations. Therefore, only data from the remaining seven stations were used in the subsequent analysis.

Because the monitoring records were available at the group level rather than the species level, this study did not further determine the species-specific recorded insect status or economic significance of individual recorded insects. Accordingly, “Coleoptera” and “Lepidoptera” refer to the insect groups recorded by the monitoring system, and the analyses focus on their recorded abundance and temporal dynamics rather than species-specific recorded insect abundance or economic damage.

#### 2.2.2. Meteorological Data

Meteorological data were obtained from the China Meteorological Administration Land Data Assimilation System (CLDAS-V2.0) near real-time product dataset (https://data.cma.cn/data/cdcdetail/dataCode/NAFP_CLDAS2.0_NRT.html (accessed on 13 August 2026)). This dataset integrates ground-based automatic weather station observations, satellite remote sensing data, and numerical weather prediction data, and provides multiple meteorological variables including temperature, humidity, wind speed, radiation, land surface temperature, and soil meteorological information. The spatial resolution is 0.0625° × 0.0625°, and the temporal resolution is 1 h.

In this study, daily scale meteorological data from 1 December 2021 to 30 September 2023 were used. The original NetCDF format data were processed through rasterization, and meteorological variables corresponding to each recorded insect monitoring station were extracted based on their spatial locations.

Previous studies have shown that meteorological factors such as temperature, humidity, wind speed, and solar radiation are important environmental drivers affecting insect development, activity, population dispersal, and temporal dynamics. Based on the relationships between forest-associated insects and climatic factors reported in previous studies, 2 m air temperature, 2 m specific humidity, 10 m wind speed, and shortwave radiation were selected as environmental variables. These measurement heights correspond to the standard near-surface meteorological variables provided by the CLDAS-V2.0 dataset and were therefore retained as consistent indicators of the ambient atmospheric conditions at the monitoring sites. These variables were matched with the recorded insect monitoring data from the same period to construct the environmental dataset for recorded insect prediction models. In addition, 95% confidence intervals of Pearson correlation coefficients were calculated using Fisher’s z-transformation to quantify the uncertainty of the correlation estimates.

### 2.3. Data Preprocessing

**Figure 2:**
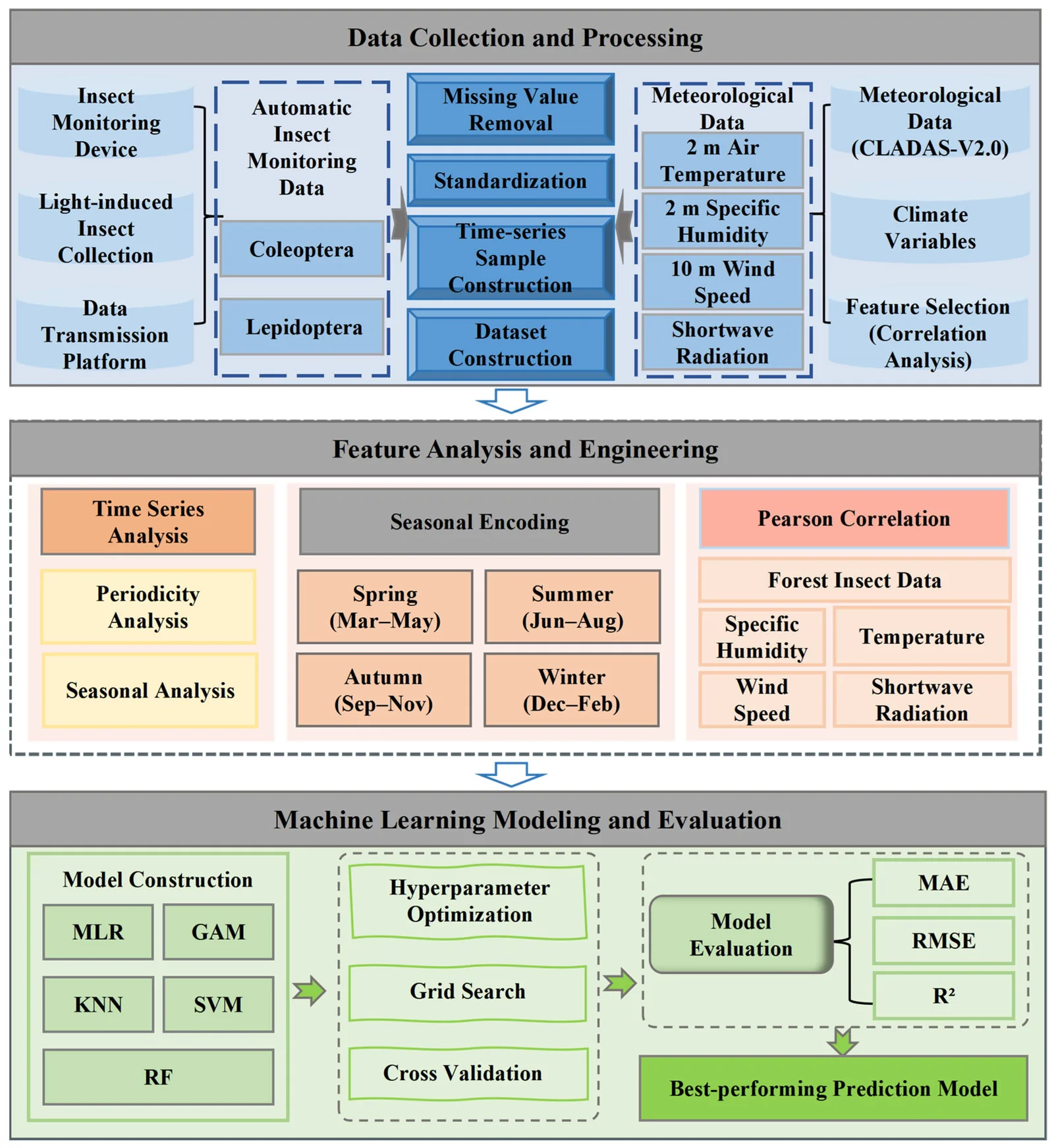
Research framework.

The overall technical workflow of this study is shown in Figure 2.

#### 2.3.1. Data Cleaning and Standardization

To reduce the impact of differences in the units and scales of meteorological variables on model training, StandardScaler was used to standardize 2 m air temperature, 2 m specific humidity, 10 m wind speed, and shortwave radiation. After standardization, each variable had a mean of 0 and a standard deviation of 1. Samples with missing values were removed directly, and no interpolation was applied in order to avoid the influence of continuous missing data on model performance. All data preprocessing procedures were implemented using the Scikit-learn version 1.0.2 in Python.

#### 2.3.2. Seasonal Feature Encoding

To improve the ability of the model to represent seasonal variation in recorded insect abundance, seasonal information was included as an input feature and was encoded using the One-Hot encoding method. Seasons were defined based on months: March to May as spring, June to August as summer, September to November as autumn, and December to February of the following year as winter. The seasonal variable was encoded using the get_dummies function in Python to achieve numerical representation. After encoding, each season was represented by a binary variable, where a value of 1 indicated that the sample belonged to the corresponding season, and 0 indicated otherwise. The One-Hot encoding scheme for seasonal variables is shown in Table 1.

#### 2.3.3. Dataset Partitioning

After standardization and seasonal feature encoding, 2 m air temperature, 2 m specific humidity, 10 m wind speed, shortwave radiation, and seasonal variables were used as input features, while the daily abundance of Coleoptera and Lepidoptera was used as the prediction target to construct the recorded insect prediction dataset. The dataset was initially divided using the train_test_split function in Scikit-learn, with 80% of the samples used for model training and 20% for internal evaluation. Considering that random partitioning may not fully represent the temporal transferability of ecological prediction models, an additional independent-year validation was conducted. Specifically, data from 2021 to 2022 were used for model training, while data from 2023 were completely excluded during model development and used as an independent validation dataset. Under the random partitioning strategy, each monitoring station contained approximately 510 training samples and 128 validation samples.

### 2.4. Model Development

#### 2.4.1. Correlation Analysis of Climatic Factors

To investigate the relationships between forest recorded insects and meteorological factors, Pearson correlation analysis was conducted between recorded insect abundance and meteorological variables. Considering that recorded insect abundance showed seasonal variation, Pearson correlation analysis was used to explore the overall associations between recorded insect abundance and climatic factors rather than to infer causal effects. Significance tests were also performed to evaluate the statistical significance of these associations.

However, Pearson correlation only describes the linear association between individual variables and recorded insect abundance and does not account for nonlinear relationships or interactions among predictors. Therefore, a Random Forest-based permutation importance analysis was further conducted to quantify the relative contribution of the predictors to model prediction within a nonlinear modeling framework.

#### 2.4.2. Prediction of Recorded Insect Abundance Based on Multiple Machine Learning Models

Five prediction models, including Multiple Linear Regression (MLR), Generalized Additive Model (GAM), K-Nearest Neighbors (KNN), Support Vector Regression (SVR), and Random Forest (RF), were developed to compare the applicability of different machine learning methods for predicting Coleoptera and Lepidoptera. The input variables included 2 m air temperature, 2 m specific humidity, 10 m wind speed, shortwave radiation, and seasonal factors, while the daily abundance of Coleoptera and Lepidoptera was used as the prediction target. Because some meteorological variables may exhibit inherent correlations in natural systems, potential multicollinearity among predictors was considered during model interpretation. The machine learning models used in this study were primarily developed for prediction rather than for estimating the independent effects of individual variables. Seasonal variables were represented using One-Hot encoded data, whereas continuous meteorological variables were represented using standardized data.

1. Multiple Linear Regression (MLR)

Multiple Linear Regression (MLR) is a classical statistical regression method that establishes linear relationships between multiple explanatory variables and a response variable. The mathematical expression of the model is shown in Equation (1):

(1) $${\hat{y}}_{i}\;=\;\omega_{0}+\sum_{j=1}^{n}\omega_{j}x_{\mathrm{ij}}$$

where ${\hat{y}}_{i}$ is the predicted recorded insect abundance of the i-th sample, $\omega_{0}$ is the intercept term, $\omega_{j}$ is the regression coefficient corresponding to the j-th explanatory variable, $x_{ij}$ is the value of the j-th explanatory variable for the i-th sample, and n is the number of explanatory variables.

The parameters of the MLR model were estimated using the least squares method, which fits the model by minimizing the sum of squared residuals between observed and predicted values. Because the MLR model was used primarily as a baseline prediction model rather than for causal inference, the estimated coefficients were interpreted mainly as model parameters for prediction rather than as independent effects of individual explanatory variables. Owing to its simple structure and good interpretability, MLR is commonly used as a baseline model for evaluating the predictive performance of nonlinear models.

2. Support Vector Regression (SVR)

The Support Vector Regression (SVR) model was used to predict recorded insect abundance. By using kernel functions, the predictor space was mapped into a higher dimensional feature space to characterize the nonlinear relationships between meteorological factors and recorded insect abundance.

SVR can effectively learn complex nonlinear features under limited sample conditions while maintaining a balance between model complexity and prediction error. Therefore, it has been widely applied in ecological and environmental prediction studies. In this study, the Radial Basis Function (RBF) kernel was adopted to construct the SVR model, and GridSearchCV was used to optimize the penalty parameter (C) and kernel parameter (γ) to obtain the best prediction performance.

3. Random Forest (RF)

Random Forest (RF) is a nonparametric machine learning method based on ensemble learning. It generates multiple training subsets through bootstrap sampling with replacement and constructs multiple decision trees for ensemble prediction. For regression problems, the final output of the Random Forest model is calculated as the average prediction of all decision trees:

(2) $$\hat{y}=\frac{1}{N}\sum_{i=1}^{N}T_{i}(x)$$

where $\hat{y}$ is the final predicted value, N is the number of decision trees, and $T_{i}\left(x\right)$ is the prediction generated by the i-th decision tree.

Random Forest can automatically learn complex nonlinear relationships and higher-order interactions among variables, while exhibiting strong robustness to outliers and multicollinearity. Considering that the occurrence of Coleoptera and Lepidoptera is characterized by pronounced nonlinear patterns and coupled effects of multiple meteorological factors, Random Forest was employed to develop the prediction model. GridSearchCV was used to optimize model parameters, including the number of decision trees (n_estimators), maximum tree depth (max_depth), minimum number of samples required for splitting (min_samples_split), minimum number of samples required at a leaf node (min_samples_leaf), and maximum feature proportion (max_features).The parameter search ranges were set as follows: n_estimators ranged from 10 to 200 with a step size of 1; max_depth ranged from 10 to 30 with a step size of 3; min_samples_split ranged from 2 to 10 with a step size of 1; min_samples_leaf ranged from 2 to 10 with a step size of 1; and max_features ranged from 0.1 to 1 with a step size of 0.1. A 10-fold cross-validation strategy was used within the training dataset, and R^2^ was used as the scoring metric. The parameter combination with the highest mean cross-validation R^2^ was selected as the optimal configuration.

In addition, to further compare the predictive performance of different model types, K-Nearest Neighbors (KNN) and Generalized Additive Model (GAM) were included as benchmark models. KNN performs prediction based on neighborhood relationships in the feature space, whereas GAM characterizes nonlinear relationships between explanatory and the response variable through smoothing functions. The MLR, KNN, SVR, and RF models were implemented using the Scikit-learn library, while the GAM was implemented using the pyGAM library. All models were trained and evaluated using the same training and validation datasets. For KNN and SVR, GridSearchCV with 5-fold cross-validation was used for hyperparameter optimization within the training dataset, and the optimal parameter combination was selected based on the highest mean cross-validation R^2^. MLR was fitted directly using the least-squares method without hyperparameter tuning.

#### 2.4.3. Model Evaluation Metrics

Mean Absolute Error (MAE), Root Mean Square Error (RMSE), and the coefficient of determination (R^2^) were used to evaluate model performance. MAE and RMSE were used to measure the differences between predicted and observed values, whereas R^2^ was used to assess the ability of the model to explain the variation in the response variable. Lower MAE and RMSE values and a higher R^2^ value indicate better model performance.

#### 2.4.4. Feature Importance Analysis

To improve the interpretability of the Random Forest prediction models, permutation importance analysis was conducted for the optimized Random Forest models of Coleoptera and Lepidoptera, respectively. Permutation importance evaluates the contribution of each predictor by randomly shuffling its values while keeping the other predictors unchanged and measuring the resulting decrease in model performance. In this study, the coefficient of determination (R^2^) was used as the performance metric. A larger decrease in R^2^ after permutation indicated a greater contribution of the corresponding predictor to the predictive performance of the Random Forest model.

The permutation procedure was repeated 50 times for each predictor to reduce the influence of random variation, and the mean permutation importance was calculated. To facilitate comparison among predictors, the permutation importance values were further normalized to obtain their relative importance (%). The analysis was conducted separately for Coleoptera and Lepidoptera using the corresponding optimized Random Forest models and the 20% validation dataset. The relative importance of meteorological and seasonal predictors was then compared to identify the major predictors contributing to recorded insect abundance prediction.

## 3. Results

### 3.1. Temporal Variation Characteristics of Coleoptera and Lepidoptera

#### 3.1.1. Periodic Characteristics of Coleoptera and Lepidoptera

**Figure 3:**
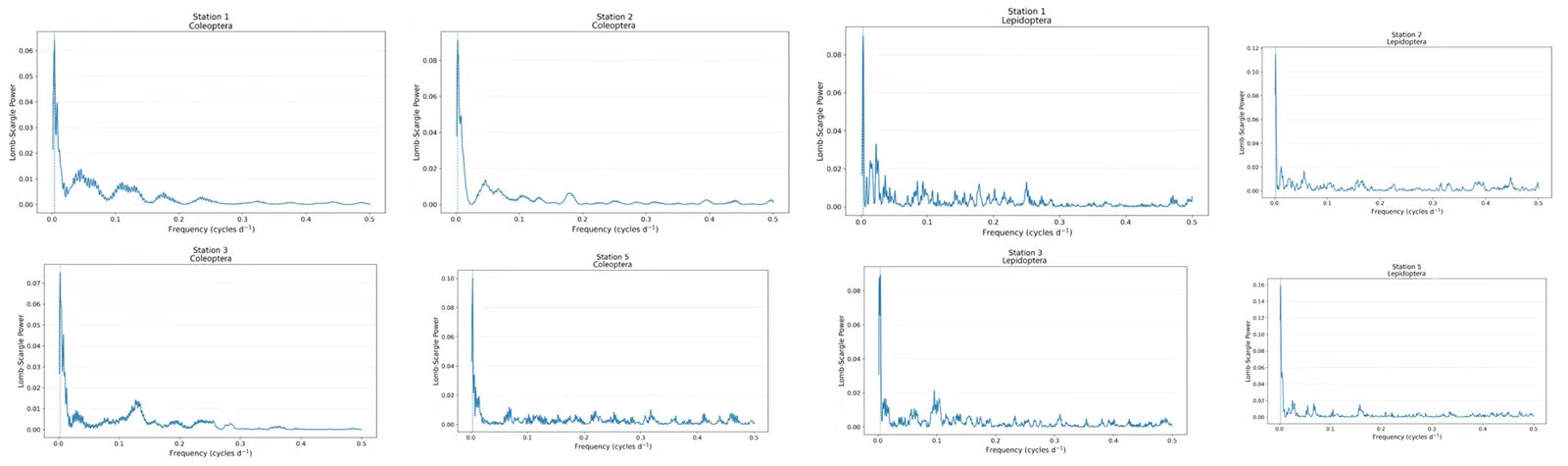

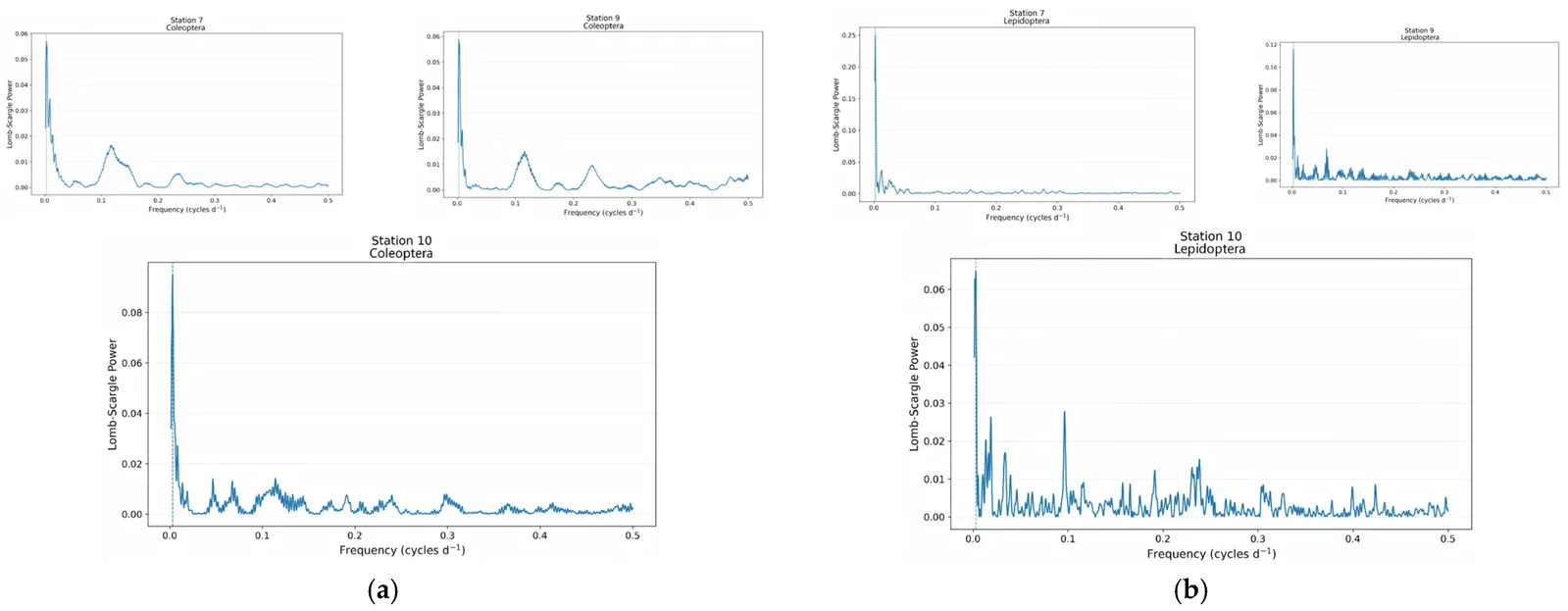
Lomb–Scargle periodograms of the recorded abundance of Coleoptera and Lepidoptera across monitoring stations in Rui’an City. (**a**) Coleoptera; (**b**) Lepidoptera.

The time series of both recorded insect groups were examined using the Lomb–Scargle periodogram to accommodate unevenly sampled data, and exhibited periodic fluctuations, although the strength of periodicity differed considerably between them (Figure 3a,b). Coleoptera showed relatively stable periodic variation. However, unlike the previous spectral analysis, the dominant peaks in the Lomb–Scargle spectra occurred at the lowest frequencies examined for all three stations, with Station 2 showing the highest power (0.08), followed by Station 3 (0.07) and Station 1 (0.06). The spectral power declined rapidly with increasing frequency, and no significant peaks were detected beyond 0.20 cycles d^−1^, indicating that the primary signal arises from long-term trends rather than distinct short-term cycles. In contrast, the periodic structure of Lepidoptera was less evident, and only a few stations exhibited local spectral peaks. For example, sporadic low-amplitude signals appeared at a few frequencies (e.g., near 0.08–0.11 cycles d^−1^) but without consistent station-wise patterns, whereas the remaining stations did not exhibit stable periodic patterns and instead showed more dispersed fluctuation characteristics.

Both insect groups exhibited relatively high spectral values in the low frequency range, indicating the influence of trend components in the time series. In addition to the low frequency peaks, the Coleoptera spectra did not show a concentrated distribution in the 0.05–0.15 range as previously noted; instead, the power decreased monotonically from zero frequency. Lepidoptera showed only isolated, weak peaks at a few stations. Overall, Coleoptera exhibited stronger low-frequency signals than Lepidoptera, whereas neither insect group showed consistent short-term periodicity across monitoring stations.

#### 3.1.2. Seasonal Characteristics of the Two Recorded Insect Groups

**Figure 4:**
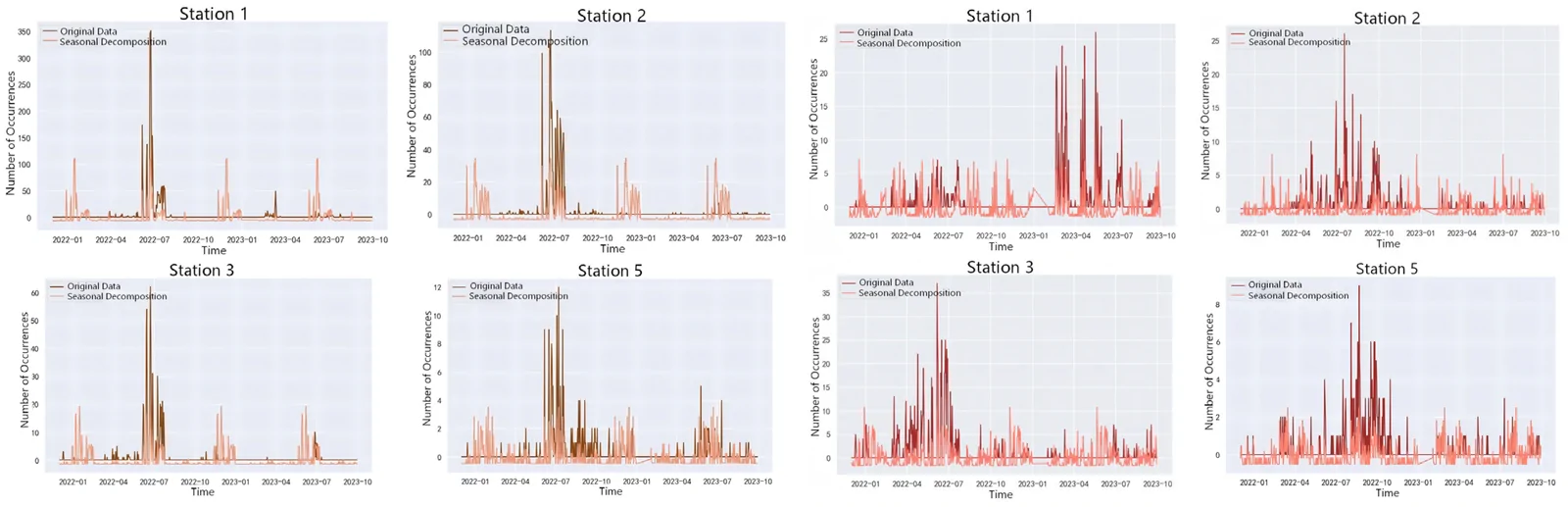

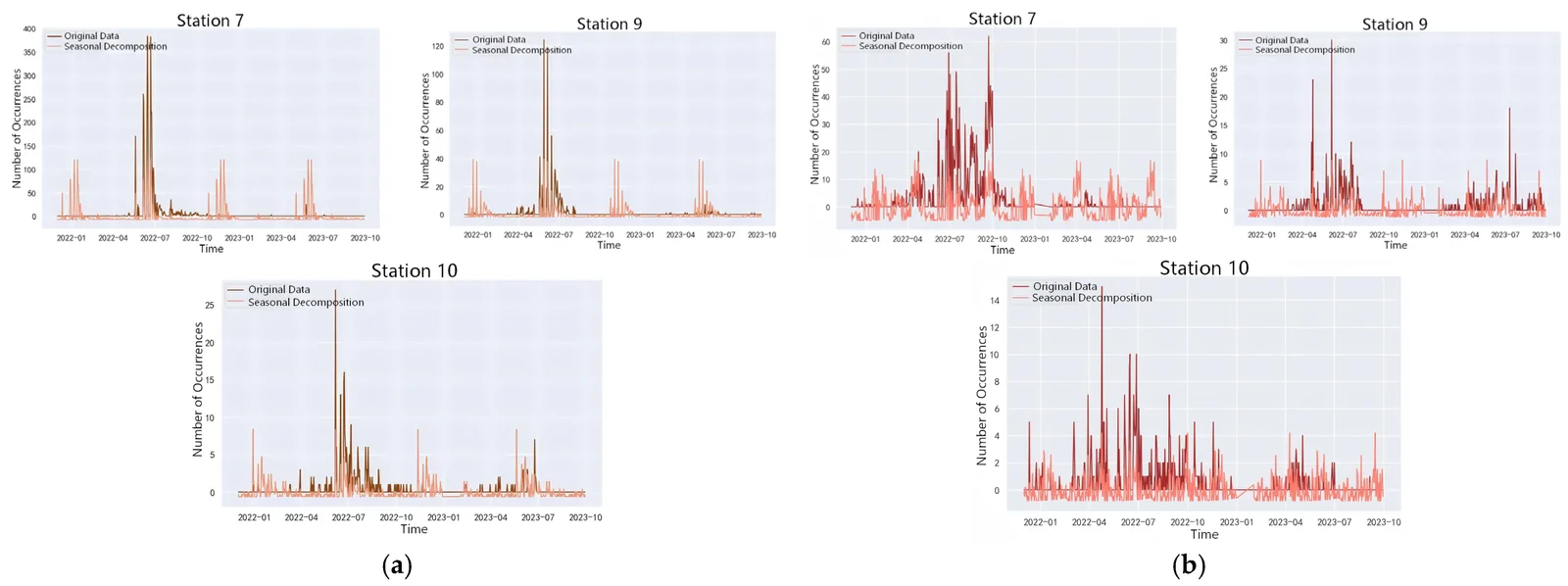
Seasonal variation in recorded abundance of Coleoptera and Lepidoptera across monitoring stations in Rui’an City. (**a**) Coleoptera; (**b**) Lepidoptera.

Both recorded insect groups exhibited distinct seasonal variation patterns, although their temporal distribution patterns differed considerably (Figure 4a,b). Coleoptera experienced a period of elevated recorded abundance during June to August 2022, with most monitoring stations reaching peak abundance during the same period. At some stations, the daily abundance exceeded 100 individuals. The seasonal decomposition results showed that the seasonal variation patterns were generally consistent among stations, with summer representing the primary period of elevated recorded abundance and exhibiting strong seasonal synchrony. In addition, some stations showed local peaks in January, indicating the presence of multiple periods of elevated recorded abundance within a year. In contrast, Station 5 exhibited relatively weak fluctuations and lower abundance, and its seasonal characteristics were less pronounced than those observed at other stations.

The overall abundance of Lepidoptera was lower than that of Coleoptera, and its temporal fluctuations were relatively moderate. However, Lepidoptera remained continuously active from April to October, exhibiting seasonal characteristics with a longer duration. At most stations, abundance generally increased during the spring and summer of 2022, while Station 1 reached a relatively high level during April to October 2023. Overall, Lepidoptera did not exhibit a pronounced concentration of recorded abundance but instead showed a seasonal fluctuation pattern that persisted for a longer period.

### 3.2. Correlation Analysis Between Recorded Insect Abundance and Climatic Factors

Both Coleoptera and Lepidoptera showed significant positive correlations with air temperature (TMP), specific humidity (SHU), and shortwave radiation (SSRA) (*p* < 0.01; Table 2), indicating that recorded insect abundance was positively associated with variations in these climatic factors. Among the two recorded insect groups, Lepidoptera exhibited generally stronger correlations with meteorological factors than Coleoptera, suggesting different association patterns between the two recorded insect groups and climatic variations. In contrast, wind speed (WIN) showed weak negative correlations with both recorded insect groups, indicating a weak negative association between wind speed and recorded insect abundance.

Correlations were also observed among the meteorological factors. The strongest correlation was found between specific humidity and air temperature (r = 0.956, *p* < 0.01), indicating a strong synchronous variation between temperature and humidity conditions in the study area. Shortwave radiation was also significantly and positively correlated with both air temperature and specific humidity. Except for wind speed, all meteorological factors showed significant correlations with recorded insect abundance, suggesting that the selected variables can effectively represent the climatic conditions associated with recorded insect abundance. The strong correlation between air temperature and specific humidity indicates potential collinearity among climatic variables.

### 3.3. Evaluation of Model Prediction Performance

To improve model performance, hyperparameters of all machine learning models were optimized using cross-validation combined with grid search. The optimized parameter combinations of the Random Forest models for the two recorded insect groups are presented in Table 3, while those of the other models are provided in Table A1, Table A2 and Table A3. For Random Forest, the optimal numbers of decision trees were 50 and 182 for Coleoptera and Lepidoptera, respectively, while the remaining parameters were identical for the two recorded insect groups. After hyperparameter optimization, the R^2^ values of the Random Forest model increased by 0.14 and 0.03 for Coleoptera and Lepidoptera, respectively, indicating improved predictive performance after parameter tuning.

The predictive performance of the different machine learning models varied considerably between the two recorded insect groups (Table 4). Under the random validation strategy, the Random Forest model achieved the best predictive performance for both recorded insect groups, showing the lowest prediction errors and the highest R^2^ values among the evaluated models. For Coleoptera, the MAE, RMSE, and R^2^ of the Random Forest model were 1.37, 4.85, and 0.82, respectively. For Lepidoptera, the corresponding values were 0.57, 1.16, and 0.84, respectively. These results indicate that the Random Forest model achieved strong predictive performance under the internal validation framework. Paired bootstrap comparisons further showed that the Random Forest model had significantly lower MAE and RMSE than the other four models for both recorded insect groups (all *p* < 0.001; Table A4).

In contrast, Multiple Linear Regression and the Generalized Additive Model showed relatively poor predictive performance, with R^2^ values below 0.25 for both recorded insect groups. This suggests that traditional linear methods were unable to adequately characterize the complex nonlinear relationships between recorded insect abundance and climatic factors. The K Nearest Neighbor model achieved moderate prediction accuracy for both insect groups, with better performance for Lepidoptera than for Coleoptera. The Support Vector Regression model performed better than Multiple Linear Regression in terms of error metrics; however, its R^2^ value remained close to zero, indicating that although it reduced some of the prediction error, it was unable to effectively explain the overall variation in recorded insect abundance.

To further evaluate the temporal transferability of the developed models, an independent-year validation was conducted using data from 2023, while data from 2021 to 2022 were used for model training. The results are presented in Table A5. Compared with the random validation results, model performance generally decreased under independent-year validation, particularly in terms of R^2^ values. This indicates that the models exhibited reduced generalization ability when applied to an unseen year. The discrepancy between random validation and independent-year validation suggests that the performance obtained from random partitioning may be relatively optimistic. This is particularly relevant because random partitioning allows samples from the same monitoring stations and adjacent periods to occur in both the training and validation sets, which may reduce the difficulty of temporal prediction. The lower performance under independent-year validation provides a more stringent assessment of temporal generalization and indicates that the models may not fully capture interannual variation in recorded insect population dynamics. Therefore, the high R^2^ values obtained under random validation should be interpreted as internal predictive performance rather than as evidence of robust long-term generalization. The discrepancy may also be attributed to the strong temporal variability of recorded insect abundance, differences in population dynamics between years, and the limited observation period. Therefore, although Random Forest showed superior performance under the random validation strategy, its long-term predictive stability requires further evaluation using longer-term monitoring datasets.

Overall, Random Forest exhibited the best predictive performance under the random validation framework and showed strong capability in capturing nonlinear relationships between climatic factors and recorded insect abundance. However, the reduced performance observed in independent-year validation suggests that temporal generalization remains challenging for short-term prediction of forest insect abundance. Future studies incorporating longer-term observations and additional ecological variables are therefore needed to improve model robustness and transferability.

### 3.4. Feature Importance of Random Forest Models

**Figure 5:**
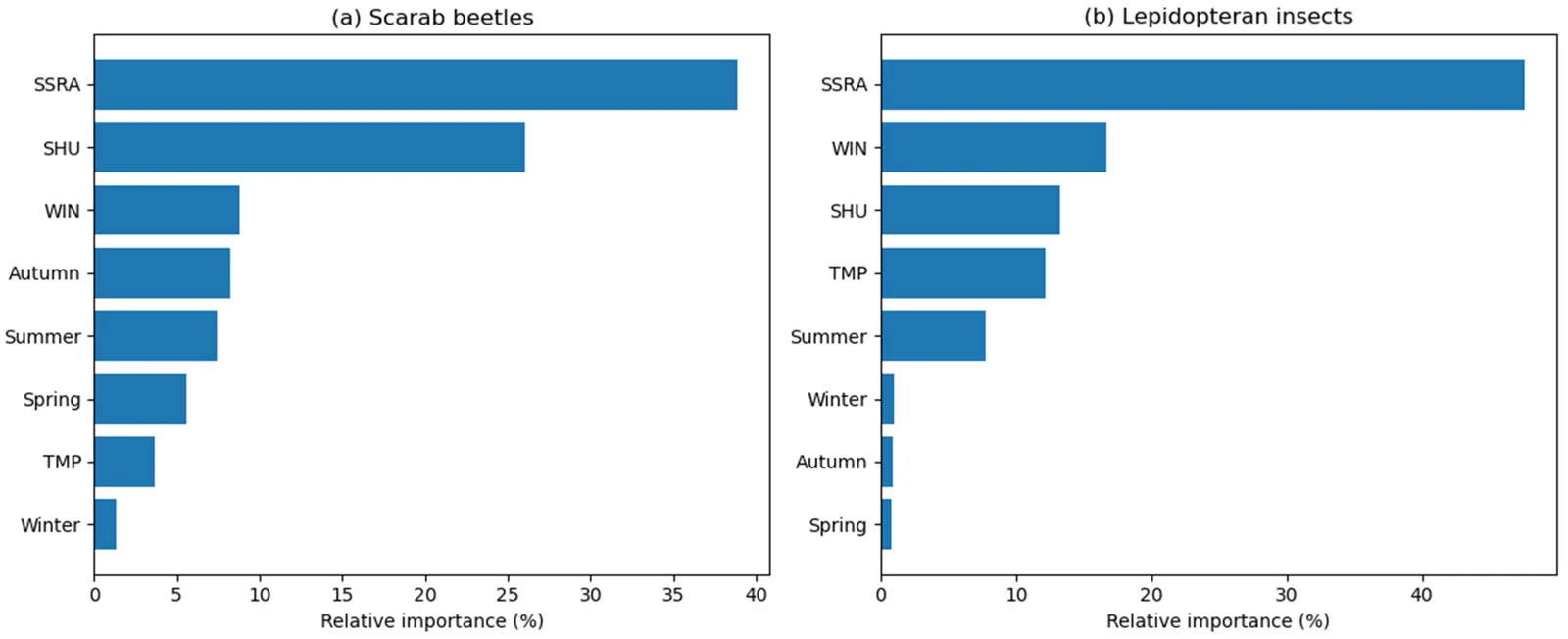
Relative importance of predictors in the Random Forest models for (**a**) Coleoptera and (**b**) Lepidoptera.

The permutation importance analysis revealed distinct differences in the relative contributions of the predictors to the Random Forest models for the two recorded insect groups (Figure 5). Shortwave radiation (SSRA) was the most important predictor for both Coleoptera and Lepidoptera, although the relative contributions of the other predictors differed between the two recorded insect groups.

For Coleoptera, SSRA showed the highest relative importance, followed by specific humidity (SHU) and wind speed (WIN). Seasonal variables, particularly autumn and summer, also contributed to the model prediction, whereas air temperature (TMP) and winter showed relatively lower importance. This indicates that shortwave radiation and specific humidity were the major predictors captured by the Random Forest model for scarab beetle abundance.

For Lepidoptera, SSRA likewise exhibited the highest relative importance. Wind speed showed the second-highest contribution, followed by specific humidity and air temperature. The seasonal variables showed relatively low contributions compared with the major meteorological predictors. The different ranking patterns between the two recorded insect groups indicate that the Random Forest models captured distinct predictor contribution structures for Coleoptera and Lepidoptera.

Overall, shortwave radiation consistently showed the greatest relative importance for both recorded insect groups, whereas the contributions of specific humidity, wind speed, air temperature, and seasonal variables varied between the two groups. These results provide additional information beyond the Pearson correlation analysis by indicating which predictors contributed most strongly to the predictive performance of the nonlinear Random Forest models.

## 4. Discussion

This study is based on multi-site forest recorded insect monitoring data from Rui’an City. By combining meteorological factors and time series features, a forest recorded insect prediction model was developed, and the predictive performance of different machine learning methods was compared. The results show that (1) both types of forest recorded insects exhibit clear temporal variation characteristics, where Coleoptera show relatively stable short-period fluctuations and exhibit a concentrated period of elevated recorded abundance in summer, while Lepidoptera show weaker periodicity but maintain a relatively high occurrence level from April to October, indicating a longer-lasting seasonal variation pattern; (2) temperature, specific humidity, and shortwave radiation are significantly positively correlated with the occurrence of both recorded insect types, while wind speed shows a weak negative correlation, indicating that climatic conditions are closely associated with forest recorded insect dynamics; (3) different machine learning models show clear differences in predictive performance, and the Random Forest model achieves the best results for both recorded insect types, with R^2^ values of 0.82 and 0.84 for Coleoptera and Lepidoptera, respectively, indicating that the Random Forest model showed strong predictive performance under the internal validation framework. However, independent-year validation revealed reduced predictive performance when the models were applied to an unseen year, suggesting that temporal generalization remains challenging for forest recorded insect prediction.

The time series analysis of forest recorded insects shows clear differences between the two recorded insect types in the temporal dimension. Coleoptera show stable short-period fluctuations at most monitoring sites, with a main cycle of about 6–20 days, and periods of elevated recorded abundance occur mainly in summer, indicating strong seasonal characteristics and synchronization. In contrast, Lepidoptera show weaker periodicity but maintain a relatively high occurrence level from April to October, showing long duration and relatively smooth variation. This difference may be related to differences in life history characteristics, developmental rates, and reproductive rhythms between the two recorded insect types [26]. This finding is broadly consistent with previous studies showing that insect activity and abundance can exhibit strong seasonal patterns associated with temperature, resource availability, and species-specific phenology [27,28]. However, the different temporal patterns observed between the two groups in the present study also suggest that climatic effects may interact with taxon-specific biological characteristics rather than producing a uniform seasonal response across insect groups. Therefore, periodic and seasonal characteristics in recorded insect time series can reflect, to some extent, the driving effects of environmental change on recorded insect population dynamics.

Temperature, specific humidity, and shortwave radiation are significantly positively correlated with the occurrence of both recorded insect types. Notably, Lepidoptera exhibit generally stronger correlations with climatic factors than Coleoptera, indicating that the two recorded insect groups show different associations with environmental conditions. This result is consistent with previous studies showing that temperature and solar radiation can influence insect activity, development, and flight behavior [29]. However, the stronger climatic associations observed for Lepidoptera in the present study suggest that the sensitivity of recorded insect activity to climatic conditions may differ among taxonomic groups. Such differences may reflect variation in thermal requirements, developmental rates, and activity periods among insect taxa. Wind speed shows a weak negative correlation with both recorded insect types, which may reflect the potential influence of wind conditions on insect activity and dispersal processes. Therefore, the correlation results should be interpreted as overall associations rather than independent effects of individual climatic variables. The observed relationships indicate that multiple climatic variables jointly characterize the environmental conditions associated with recorded insect abundance. Interestingly, although shortwave radiation showed a weaker linear correlation with recorded abundance than some other climatic variables, permutation importance analysis identified it as the most important predictor in the Random Forest models for both recorded insect groups. This difference highlights the value of combining conventional correlation analysis with nonlinear machine-learning-based interpretation: a predictor with a relatively modest marginal linear association may still provide substantial predictive information when nonlinear relationships and interactions are considered. However, given the strong correlation between air temperature and specific humidity, feature importance should be interpreted as relative predictive contribution rather than independent causal effects.

Under the random validation strategy, the Random Forest model showed superior predictive performance for both Coleoptera and Lepidoptera. Compared with multiple linear regression, generalized additive model, K-nearest neighbors, and SVR models, the Random Forest model achieved the lowest prediction errors and the highest R^2^ values, indicating its effectiveness in capturing nonlinear relationships between meteorological factors and recorded insect abundance. Recent studies have emphasized the importance of ecological forecasting approaches for predicting insect population dynamics under changing environmental conditions, particularly the need to account for temporal structure and uncertainty in monitoring data [30]. Similar to these studies, the superior performance of Random Forest in this study may be attributed to its ensemble structure, which integrates multiple decision trees and reduces the instability of individual models, thereby improving its ability to capture complex feature relationships [31]. The relatively poor performance of MLR and GAM may be partly related to the strong correlation between temperature and specific humidity, which can reduce the stability of traditional regression-based models when predictors contain overlapping information. In contrast, Random Forest is generally less sensitive to correlated predictors because random subsets of predictors are considered at each tree split, while its tree-based structure can flexibly capture nonlinear relationships and interactions. SVR showed particularly poor performance for one prediction task, with a negative R^2^ value indicating that its predictions were worse than using the mean observed value as the predictor. This may be related to the difficulty of identifying an appropriate combination of hyperparameters for the heterogeneous and noisy monitoring data, despite grid-search optimization, as well as the sensitivity of the RBF kernel to the local structure of the predictors. These results suggest that ensemble learning models may be better suited to the complex and nonlinear relationships present in the current insect monitoring data than traditional statistical models. Importantly, the lower performance observed under independent-year validation indicates that high predictive accuracy under random partitioning does not necessarily imply reliable temporal forecasting. Model-specification uncertainty and temporal variability in environmental conditions can substantially affect predictions of future forest recorded insect abundance [30,32]. Ecological forecasting studies have also emphasized the need to account for temporal autocorrelation and to evaluate models using temporally independent predictions when assessing forecast skill [30]. Although deep learning approaches such as long short-term memory (LSTM) networks can capture long-term temporal dependencies [33], their effectiveness in ecological forecasting may depend on the availability of sufficiently long and informative time series. Therefore, although the Random Forest model demonstrated the best predictive capability under the current validation framework, future studies should incorporate longer-term monitoring datasets and explore advanced approaches, including gradient boosting algorithms and deep learning models, to further improve the robustness and temporal transferability of forest recorded insect prediction.

This study still has some limitations: (1) The time span of the data is relatively limited, and the independent-year validation results indicated that model performance decreased when predicting unseen temporal conditions. This suggests that longer-term monitoring datasets covering multiple seasonal cycles and periods of elevated recorded abundance are needed to improve model generalization and stability. Although cross-validation and hyperparameter optimization were used during model development, the high R^2^ values obtained under random validation may still be partly influenced by temporal similarity between training and validation samples. Therefore, a potential risk of overfitting and optimistic estimation of predictive performance cannot be completely excluded, highlighting the importance of temporally independent validation and longer-term monitoring data. (2) The influencing factors considered in this study mainly include meteorological variables and seasonal factors, while ecological variables such as vegetation type, land use, terrain conditions, and human disturbance were not included. In addition, the high correlation among some meteorological variables (e.g., temperature and specific humidity) may introduce multicollinearity. Although machine learning models generally have stronger tolerance to correlated predictors than traditional regression approaches, future studies could further evaluate variable selection strategies or dimensionality reduction methods to improve model interpretability. (3) This study mainly uses traditional machine learning models, and future research can further combine deep learning and spatiotemporal modeling methods, such as long short-term memory (LSTM) or graph neural network models, to better capture complex spatiotemporal dependencies. Moreover, although Pearson correlation analysis was used to explore overall relationships between recorded insect abundance and climatic factors, seasonal patterns and temporal dependence may affect correlation estimates. Future studies incorporating time-series approaches, such as lagged climatic response analysis, are needed to further investigate delayed responses of recorded insect populations to climatic variations.

## 5. Conclusions

In summary, this study analyzed Coleoptera and Lepidoptera recorded by automatic monitoring stations to characterize the temporal variation in Rui’an City and evaluated the applicability of multiple machine learning models for recorded insect prediction. The results show that Coleoptera exhibit clear periodic and seasonal variation characteristics, while Lepidoptera show relatively persistent seasonal fluctuations. Temperature, specific humidity, and shortwave radiation showed significant positive associations with recorded insect abundance. The Random Forest model achieved the best performance under the random validation strategy by effectively capturing nonlinear relationships between climatic factors and recorded insect abundance. However, independent-year validation revealed reduced predictive performance under unseen temporal conditions, highlighting the need for longer monitoring records and additional environmental variables to improve model robustness and temporal generalization. These findings provide useful insights for forest recorded insect monitoring and provide a basis for further research on data-driven prediction of recorded insect abundance.

## Figures and Tables

**Table 1:** One-Hot encoding scheme for seasonal variables used as model input features.

Month	Spring	Summer	Autumn	Winter
1, 2, 12	0	0	0	1
3, 4, 5	1	0	0	0
6, 7, 8	0	1	0	0
9, 10, 11	0	0	1	0

Note: Seasons were defined according to calendar months: spring = March–May, summer = June–August, autumn = September–November, and winter = December–February. A value of 1 indicates membership in the corresponding season, whereas 0 indicates otherwise.

**Table 2:** Pearson correlation matrix between recorded insect abundance and climatic factors, including correlation coefficients and 95% confidence intervals.

Variable	Mean	Standard Deviation	Coleoptera	Lepidoptera	SHU	WIN	SSRA	TMP
Coleoptera	2.346	17.733	1					
Lepidoptera	1.260	4.253	0.285 (0.258, 0.312) **	1				
SHU	0.012	0.006	0.137 (0.108, 0.166) **	0.168 (0.140, 0.196) **	1			
WIN	0.914	0.429	−0.032 (−0.061, 0.003) *	−0.042 (−0.071, −0.013) **	−0.034 (−0.063, −0.005) *	1		
SSRA	162.299	82.227	0.111 (0.082, 0.140) **	0.212 (0.184, 0.240) **	0.350 (0.323, 0.376) **	0.070 (0.041, 0.099) **	1	
TMP	19.254	7.860	0.127 (0.098, 0.156) **	0.188 (0.160, 0.216) **	0.956 (0.955, 0.957) **	0.033 (0.004, 0.062) *	0.481 (0.457, 0.505) **	1

Note: Values are Pearson correlation coefficients with 95% confidence intervals in parentheses. * and ** indicate statistical significance at the 0.05 and 0.01 levels, respectively, based on two-tailed tests. SHU, specific humidity; WIN, wind speed; SSRA, shortwave radiation; TMP, air temperature.

**Table 3:** Optimal hyperparameter configurations of the Random Forest models for predicting recorded abundance of Coleoptera and Lepidoptera.

Parameter	Coleoptera	Lepidoptera
n_estimators	50	182
max_depth	10	10
min_samples_split	9	9
min_samples_leaf	9	9
max_features	0.1	0.1

Note: The optimal hyperparameters were selected using GridSearchCV with 10-fold cross-validation within the training dataset, using R^2^ as the scoring metric. n_estimators, number of decision trees; max_depth, maximum tree depth; min_samples_split, minimum number of samples required to split an internal node; min_samples_leaf, minimum number of samples required at a leaf node; max_features, proportion of features considered when splitting a node.

**Table 4:** Predictive performance of five machine learning models for Coleoptera and Lepidoptera under random validation.

Recorded Insect Category	Metric	MLR	KNN	RF	SVR	GAM
Coleoptera	MAE	4.23	2.84	1.37	2.39	4.55
	RMSE	12.40	10.85	4.85	13.03	11.88
	R^2^	0.08	0.30	0.82	−0.01	0.16
Lepidoptera	MAE	1.74	1.23	0.57	1.24	1.60
	RMSE	3.09	2.55	1.16	3.34	2.87
	R^2^	0.12	0.36	0.84	−0.01	0.23

Note: Values represent predictive performance on the 20% validation dataset under the random partitioning strategy. MAE, mean absolute error; RMSE, root mean square error; R^2^, coefficient of determination. Lower MAE and RMSE values and higher R^2^ values indicate better predictive performance. MLR, multiple linear regression; KNN, K-nearest neighbors; RF, Random Forest; SVR, support vector regression; GAM, generalized additive model.

## Data Availability

The data presented in this study are available upon request from the corresponding author.

## Appendix A

**Table A1:** Estimated coefficients of multiple linear regression models for two recorded insect types.

Variable	Coleopteras Coefficient	Coleopteras Intercept	Lepidoptera Coefficient	Lepidoptera Intercept
X1	1.64	2.10	0.51	1.24
X2	1.17		−0.65	
X3	−0.39		0.01	
X4	−1.87		1.19	
X5	−1.04		−0.10	
X6	−1.75		−0.03	
X7	4.77		−0.01	
X8	−1.98		0.14	

Note: X1–X4 represent four meteorological factors (shortwave radiation, specific humidity, wind speed, and temperature), and X5–X8 represent seasonal factors (spring, summer, autumn, and winter).

**Table A2:** Optimal parameter values of K-nearest neighbors regression models for two recorded insect types.

Parameter	Coleoptera	Lepidoptera
n_neighbors	9	9
weights	uniform	uniform
algorithm	auto	auto
leaf_size	20	20
metric	manhattan	minkowski

**Table A3:** Optimal parameter values of support vector machine regression models for two recorded insect types.

Parameter	Coleoptera	Lepidoptera
Kernel	rbf	rbf
C	10	10
epsilon	0.3	0.3

**Table A4:** Paired bootstrap comparisons of the Random Forest model against alternative predictive models.

Insect Category	Comparison	MAE Difference	MAE 95% CI	MAE *p*-Value	RMSE Difference	RMSE 95% CI	RMSE *p*-Value
Coleoptera	RF vs. MLR	−2.794	[−3.147, −2.472]	<0.001	−10.940	[−13.688, −7.986]	<0.001
	RF vs. KNN	−1.887	[−2.202, −1.592]	<0.001	−9.212	[−10.985, −7.455]	<0.001
	RF vs. SVR	−1.014	[−1.391, −0.666]	<0.001	−11.397	[−14.254, −8.337]	<0.001
	RF vs. GAM	−3.150	[−3.490, −2.838]	<0.001	−10.749	[−13.447, −7.864]	<0.001
Lepidoptera	RF vs. MLR	−1.198	[−1.273, −1.128]	<0.001	−2.544	[−2.828, −2.259]	<0.001
	RF vs. KNN	−0.837	[−0.905, −0.770]	<0.001	−2.122	[−2.312, −1.936]	<0.001
	RF vs. SVR	−0.642	[−0.735, −0.554]	<0.001	−2.824	[−3.136, −2.512]	<0.001
	RF vs. GAM	−1.163	[−1.236, −1.094]	<0.001	−2.483	[−2.758, −2.208]	<0.001

Note: Differences were calculated as RF minus the corresponding alternative model. Negative values indicate lower prediction errors for RF. The 95% confidence intervals were obtained by paired bootstrap resampling of the validation-set prediction errors.

**Table A5:** Performance of five machine learning models based on independent-year validation.

Recorded Insect Category	Metric	MLR	KNN	RF	SVR	GAM
Coleoptera	MAE	6.32	4.82	6.13	1.07	9.03
	RMSE	11.48	11.61	11.84	2.36	18.11
	R^2^	−35.82	−36.60	−38.14	−0.55	−90.55
Lepidoptera	MAE	2.71	2.66	2.54	2.01	2.78
	RMSE	4.80	5.19	4.63	4.31	4.94
	R^2^	−3.76	−4.56	−3.43	−2.83	−4.03

## References

[1] Huang J., Zhu S., Li Y., Yin Y., Wu J., Lv Y., Fang G. (2025). Considering compound drought and heatwave events significantly improve forest pest occurrence prediction. Pest Manag. Sci..

[2] Chagnon C., Dumont S., Morin-Bernard A., Jactel H., Achim A., Moreau G. (2025). Potential of thinning to increase forest resilience and resistance to drought, pest, windstorm and fire: A meta-analysis. For. Ecol. Manag..

[3] Masson-Delmotte V., Zhai P., Pörtner H.-O., Roberts D., Skea J., Shukla P.R., Pirani A., Moufouma-Okia W., Péan C., Pidcock R., Intergovernmental Panel on Climate Change (IPCC) (2018). Summary for Policymakers. Global Warming of 1.5°C: An IPCC Special Report on the Impacts of Global Warming of 1.5°C Above Pre-Industrial Levels and Related Global Greenhouse Gas Emission Pathways, in the Context of Strengthening the Global Response to the Threat of Climate Change, Sustainable Development, and Efforts to Eradicate Poverty.

[4] Navarro-Cerrillo R.M., Ariza-Salamanca A.J. (2024). Uncovering hazards and adaptive capacity: A comprehensive risk assessment study in three conservation areas in Spain. Forest Ecol. Manag..

[5] McTaggart E., Megiddo I., Kleczkowski A. (2023). The effect of pests and pathogens on forest harvesting regimes: A bioeconomic model. Ecol. Econ..

[6] Hesabi A., Alavi S.J., Esmailzadeh O. (2025). From data to action: MaxEnt-based conservation planning for Buxus hyrcana Pojark in the Hyrcanian forest. Environ. Sustain. Ind..

[7] Yoon S., Kim S.H., Lee W.H. (2026). Spatial prediction of Melanoplus differentialis using an ensemble of multiple species distribution models. Insect Conserv. Diver.

[8] Sun X., Siemann E., Liu Z., Wang Q., Wang D., Huang W., Zhang C., Ding J. (2019). Root-feeding larvae increase their performance by inducing leaf volatiles that attract above-ground conspecific adults. J. Ecol..

[9] Xu T., Wu X., Luo Z., Tang L., Gao J., Zang L. (2024). Light intensity differentially mediates the life cycle of lepidopteran leaf feeders and stem borers. Pest Manag. Sci..

[10] Weiss M., Kozel P., Zapletal M., Hauck D., Prochazka J., Benes J., Cizek L., Sebek P. (2021). The effect of coppicing on insect biodiversity. Small-scale mosaics of successional stages drive community turnover. For. Ecol. Manag..

[11] Guo Q., Li X., Du Y. (2026). Distribution and seasonal dynamics of two distinct sex pheromone types in Cnaphalocrocis medinalis moths across China. Pest Manag. Sci..

[12] Pokorny S.W., Aukema B.H., Raffa K.F., Carroll A.L. (2025). Population phase-dependent niche requirements may limit outbreaks of the range-expanding mountain pine beetle in the western boreal forest. For. Ecol. Manag..

[13] Xavier R.G., Martins F.B., Dias C.G. (2026). Integrated Climate, pest, and Disease Risk Indicators for Pinus Plantations Under Current and Future Climates. Earth Syst. Environ..

[14] Zhao J., Wang M., Cai D., Wu L., Ji X., Ding Q., Wang F., Wang M. (2025). Spatiotemporal Changes of Pine Caterpillar Infestation Risk and the Driving Effect of Habitat Factors in Northeast China. Remote Sens..

[15] Tan M., Meng L., Du S., He Z., Zhang Q., Chen S., Yan S., Jiang D. (2026). An emerging risk to pest biocontrol: Polyethylene microplastic affects the efficacy of Arma chinensis and Beauveria bassiana against Hyphantria cunea. J. Hazard. Mater..

[16] Mo X., Zhao X., Zhao J., Huang J., Fang G. (2025). Risk prediction of pine wilt disease based on graphical convolutional network in China. Pest Manag. Sci..

[17] Gougherty A.V., Clipp H.L., Prasad A.M., Peters M.P., Matthews S.N. (2025). Integrating disturbance to improve our understanding of range-wide patterns in tree species abundance and demography. For. Ecol. Manag..

[18] Qi T., Dong Y., Li X., Zhao M., Huang W. (2024). Fall armyworm habitat analysis in Africa with multi-source earth observation data. Comput. Electron. Agr..

[19] Diem J.E., Bailey K., Muleme J., Ramprasad V., Sullivan K., Hartter J., Mutegeki R., Venier M., Rothman J.M., Namusisi S. (2026). Pesticide use and climate-driven pest pressure in smallholder agriculture in equatorial Africa. J. Environ. Manag..

[20] Brookhouse M.T., Farrow R., Meyer J., McDougall K., Ward-Jones J., Wright G.T. (2024). Incidence and severity of Phoracantha-induced decline within high-elevation eucalypt woodlands are strongly associated with elevation and land management. For. Ecol. Manag..

[21] Soja A.J., Tchebakova N.M., French N.H., Flannigan M.D., Shugart H.H., Stocks B.J., Sukhinin A.I., Parfenova E.I., Chapin F.S., Stackhouse P.W. (2007). Climate-induced boreal forest change: Predictions versus current observations. Glob. Planet. Change.

[22] Rincon D.F., Esch E.D., Gutierrez-Illan J., Tesche M., Crowder D.W. (2024). Predicting insect population dynamics by linking phenology models and monitoring data. Ecol. Model..

[23] Singh S., Parmar K.S., Singh S. (2026). The role of artificial neural networks in mathematical modeling: From single architecture to hybrid frameworks in environmental system. Appl. Soft Comput..

[24] Haynes K.J., Pureswaran D.S., Allstadt A.J., Walter J.A., DeMerchant I., Liebhold A.M. (2026). Meteorological versus spatial drivers of the spatial synchrony of forest insect pest outbreaks in North America. Oikos.

[25] Santiago-García W. (2025). Regression analysis and artificial neural networks for predicting pine species volume in community forests. Ecol. Inform..

[26] Dong L., Yang X., Jin X., Liu X., Gao M., Fang J. (2026). Life History Traits and Developmental Duration of the Yellow Coster Telchinia issoria Hübner, 1819 (Lepidoptera: Nymphalidae) Under Laboratory Conditions. Insects.

[27] Solomon M.E. (1957). Dynamics of Insect Populations. Annu. Rev. Entomol..

[28] Yamamura K., Yokozawa M., Nishimori M., Ueda Y., Yokosuka T. (2006). How to analyze long-term insect population dynamics under climate change: 50-year data of three insect pests in paddy fields. Popul. Ecol..

[29] Gardner A.S., Li R., Jones J., Rogers R., Townsend M., Rodriguez-Munoz R., Hopwood P.E., Maclean I.M., Tregenza T. (2024). Behavioural thermoregulation compensates for changes in solar insolation in a wild insect. Anim. Behav..

[30] Bahlai C.A. (2023). Forecasting insect dynamics in a changing world. Curr. Opin. Insect Sci..

[31] Breiman L. (2001). Random forests. Mach. Learn..

[32] Boulanger Y., Gray D.R., Cooke B.J., De Grandpré L. (2016). Model-specification uncertainty in future forest pest outbreak. Glob. Change Biol..

[33] Hochreiter S., Schmidhuber J. (1997). Long short-term memory. Neural Comput..

